# Long-Circulating Nanobody Confers Durable Prophylaxis against Severe Acute Respiratory Syndrome Coronavirus 2 Omicron Infection

**DOI:** 10.1002/anbr.202400214

**Published:** 2025-06-27

**Authors:** Geetha Jyothi Vaskuri, Gang Ye, Fan Bu, Dong Yang, Colleen B. Jonsson, Hailey Turner-Hubbard, Sydney Winecke, Alise Mendoza, Fang Li, Chalet Tan

**Affiliations:** Department of Pharmaceutical Sciences, University of Tennessee Health Science Center, Memphis, TN 38163, USA; Department of Pharmacology, University of Minnesota Medical School, Minneapolis, MN 55455, USA; Department of Pharmacology, University of Minnesota Medical School, Minneapolis, MN 55455, USA; Regional Biocontainment Laboratory, University of Tennessee Health Science Center, Memphis, TN 38163, USA; Department of Pharmaceutical Sciences, University of Tennessee Health Science Center, Memphis, TN 38163, USA; Regional Biocontainment Laboratory, University of Tennessee Health Science Center, Memphis, TN 38163, USA; Department of Microbiology, Immunology and Biochemistry, University of Tennessee Health Science Center, Memphis, TN 38163, USA; Department of Pharmacology, University of Minnesota Medical School, Minneapolis, MN 55455, USA; Department of Pharmacology, University of Minnesota Medical School, Minneapolis, MN 55455, USA; Department of Pharmacology, University of Minnesota Medical School, Minneapolis, MN 55455, USA; Department of Pharmacology, University of Minnesota Medical School, Minneapolis, MN 55455, USA; Department of Pharmaceutical Sciences, University of Tennessee Health Science Center, Memphis, TN 38163, USA

**Keywords:** Fc engineering, FcRn, half-life extension, nanobody, pharmacokinetics, pre-exposure prophylaxis, SARS-CoV-2 Omicron

## Abstract

Breakthrough infections in vaccinated population and continuous emergence of severe acute respiratory syndrome coronavirus 2 (SARS-CoV-2) variants make it imperative to develop more efficacious medical countermeasures. Previously, an anti-SARS-CoV-2 nanobody, Nanosota-3A, that neutralizes the infection of live Omicron BA.1 with picomolar potency, is identified. Herein, Nanosota-3A is fused with the crystallizable fragment (Fc) domain of human IgG1 that contains M252Y/S254T/T256E (YTE) substitutions, named Nanosota-3A-Fc-YTE. Compared to Nanosota-3A-Fc, Nanosota-3A-Fc-YTE exhibits identical binding to the SARS-CoV-2 spike protein yet displays eightfold higher binding affinity for human neonatal Fc receptor (hFcRn) at pH 6.0. In hFcRn transgenic mice, the half-life of Nanosota-3A-Fc and Nanosota-3A-Fc-YTE is 5.1 days and 24.8 days, respectively. The mice are challenged with intranasal exposure of Omicron B.1.1.529 virus 55 days after a single dose of Nanosota-3A fusions (20 mg kg^−1^) is administered. Compared to the untreated controls, the lung viral titers in mice receiving Nanosota-3A-Fc-YTE are reduced by 104.7-fold (*p* = 0.007) with 50% of the mice free of detectable virus. By contrast, Nanosota-3A-Fc-treated mice show only 3.5-fold reduction in the viral titers (*p* = 0.41). The durable protection conferred by a single dose of Nanosota-3A-Fc-YTE administered nearly 2 months prior to the virus exposure demonstrates the promise of long-circulating nanobodies as powerful prophylactics against SARS-CoV-2.

## 1. Introduction

The COVID-19 pandemic has galvanized intense effort to prevent and treat the severe acute respiratory syndrome coronavirus 2 (SARS-CoV-2) infection.^[1]^ The ubiquity of SARS-CoV-2 in the human population only makes the development of efficacious and broadly accessible medical countermeasures more imperative.^[2]^ The SARS-CoV-2 spike protein is a critical target for therapeutic intervention, which mediates the virus entry by binding to the host angiotensin-converting enzyme 2 (ACE2). The innovative lipid nanoparticle-based mRNA vaccines that stimulate adaptive immunity against the spike protein are the mainstay combating SARS-CoV-2.^[3]^ Breakthrough infections in vaccinated population have now become a common occurrence, as the SARS-CoV-2 spike protein has undergone extensive mutations particularly in immune-elusive variants of concerns,^[4]^ underscoring the needs for updating mRNA vaccines to keep pace with the rapid evolution of spike protein.^[5]^ Neutralizing monoclonal antibodies (mAbs) that target the SARS-CoV-2 spike protein as a form of passive immunization had been an important modality, especially in patients at high risk of disease progression as well as in immune compromised patients.^[6]^ There were eight neutralizing mAbs that received the emergency use authorization (EUA) from the U.S. Food and Drug Administration (FDA). However, the rapid and continuous emergence of SARS-CoV-2 variants that escape both single mAbs and cocktails have made these mAbs obsolete and all EUAs have been revoked by the FDA since November 2023.^[7]^

Nanobodies are single-domain antibodies derived from heavy-chain-only antibodies found in camelids such as alpacas and llamas.^[8]^ With only one tenth the molecular size of conventional mAbs while maintaining exquisite antigen-binding specificity, nanobodies have numerous advantageous biological and physicochemical properties, such as improved accessibility to cryptic epitopes, increased aqueous solubility, reduced aggregation, high chemical and thermal stability, excellent tissue distribution, and the potential for intranasal and inhalation dosing. Nanobodies can be readily reengineered to combat new viral variants through structure-guided in vitro evolution, allowing for rapid adaptation to keep pace with viral mutations.^[9]^ Additionally, nanobodies can be easily bioengineered into multivalent constructs to improve the bioactivity and functionality and be produced from microbes and mammalian cell lines at much lower costs than mAbs.^[10]^ A number of nanobodies with high binding affinity for the receptor binding domain (RBD) of SARS-CoV-2 spike protein have been isolated from synthetic or immunized libraries, which exhibit potent neutralizing activity and provide effective protection against SARS-CoV-2 infection in animal models.^[11]^

Previously, we immunized an alpaca with recombinant SARS-CoV-2 spike protein and established an induced nanobody phage display library. Among the three discovered spike-binding nanobodies, Nanosota-3A was fused to human IgG1 Fc to form a homodimeric nanobody fusion.^[12]^ Binding to the SARS-CoV-2 RBD with significantly higher affinity than human ACE2 (hACE2) did, Nanosota-3A-Fc blocked the entry of SARS-CoV-2 pseudoviruses into hACE2-expressing cells, which corresponded to pre-Omicron strains (prototype, alpha, and delta), Omicron BA.1, and bat SARS2. Furthermore, Nanosota-3A-Fc neutralized the infection of live Omicron BA.1 with picomolar potency (IC_50_ = 2.3 ng mL^−1^ or 29.5 pM). Cryo-electron microscopy study revealed that Nanosota-3A bound to the SARS-CoV-2 RBD that directly blocked hACE2 binding to RBD, explaining its ability to neutralize the entry of SARS-CoV-2. Nanosota-3A could bind to the SARS-CoV-2 RBD irrespective of an open “standingup” conformation for receptor binding or a closed “lying-down” conformation for immune evasion. The antiviral efficacy of Nanosota-3A-Fc was assessed in hACE2-transgenic mice as well as the wild-type mice, 4 h post Omicron BA.1 challenge. Following an intraperitoneal injection or intranasal instillation at a dose of 10 mg kg^−1^, Nanosota-3A-Fc was found to drastically reduce the lung viral titers, demonstrating potent antiviral efficacy against Omicron BA.1 infection in therapeutic settings.^[12]^

Unmodified nanobodies undergo rapid renal excretion with a brief half-life due to their small size (14–15 kDa).^[13]^ From the pharmacokinetic standpoint, the fusion of nanobodies with the crystallizable fragment (Fc) domain of IgG not only increases the molecular size of fusion proteins above the threshold of glomerular filtration^[13]^ but also endows the interaction with the neonatal Fc receptor (FcRn) in vascular endothelial cells, resulting in improved circulation persistence.^[14]^ FcRn binds to the Fc domain with high affinity at acidic pH in the early endosomes while releasing it at neutral pH at the cell surface, salvaging IgG from intracellular degradation and transporting it across cell layers.^[14b]^ The essential role of FcRn in IgG recycling has been exploited as the basis for Fc engineering that aims to further extend the circulation half-lives of IgG mAbs. By introducing amino acid substitutions to the human IgG Fc domain via site-specific mutagenesis, the engineered Fc can bind to human FcRn (hFcRn) with increased affinity at acidic pH without affecting that at neutral pH, leading to increased recycling of IgG out of the endothelial cells and back into the circulation, which in turn prolongs the half-lives of IgG mAbs in vivo.^[15]^ A number of Fc-engineered variants with improved pH-dependent hFcRn interactions have been identified, such as the M252Y/S254T/T256E (YTE) triple–amino acid substitutions, and their clinical utilities for half-life extension have been established.^[16]^

The success of Fc engineering in IgG mAb therapeutics prompted us to investigate whether this strategy could be applied to nanobody therapeutics. To the best of our knowledge, this is the first study to develop and evaluate Fc-engineered nanobody fusion for the pharmacokinetic enhancement. Herein, we report that Fc-YTE fused Nanosota-3A not only markedly improved circulation persistence but also conferred durable prophylactic efficacy against SARS-CoV-2 Omicron infection in mice.

## 2. Results

### 2.1. Establishment of Quantitative Enzyme-Linked Immunosorbent Assay for Nanosota-3A-Fc and Nanosota-3A-Fc-YTE

**Figure 1.**
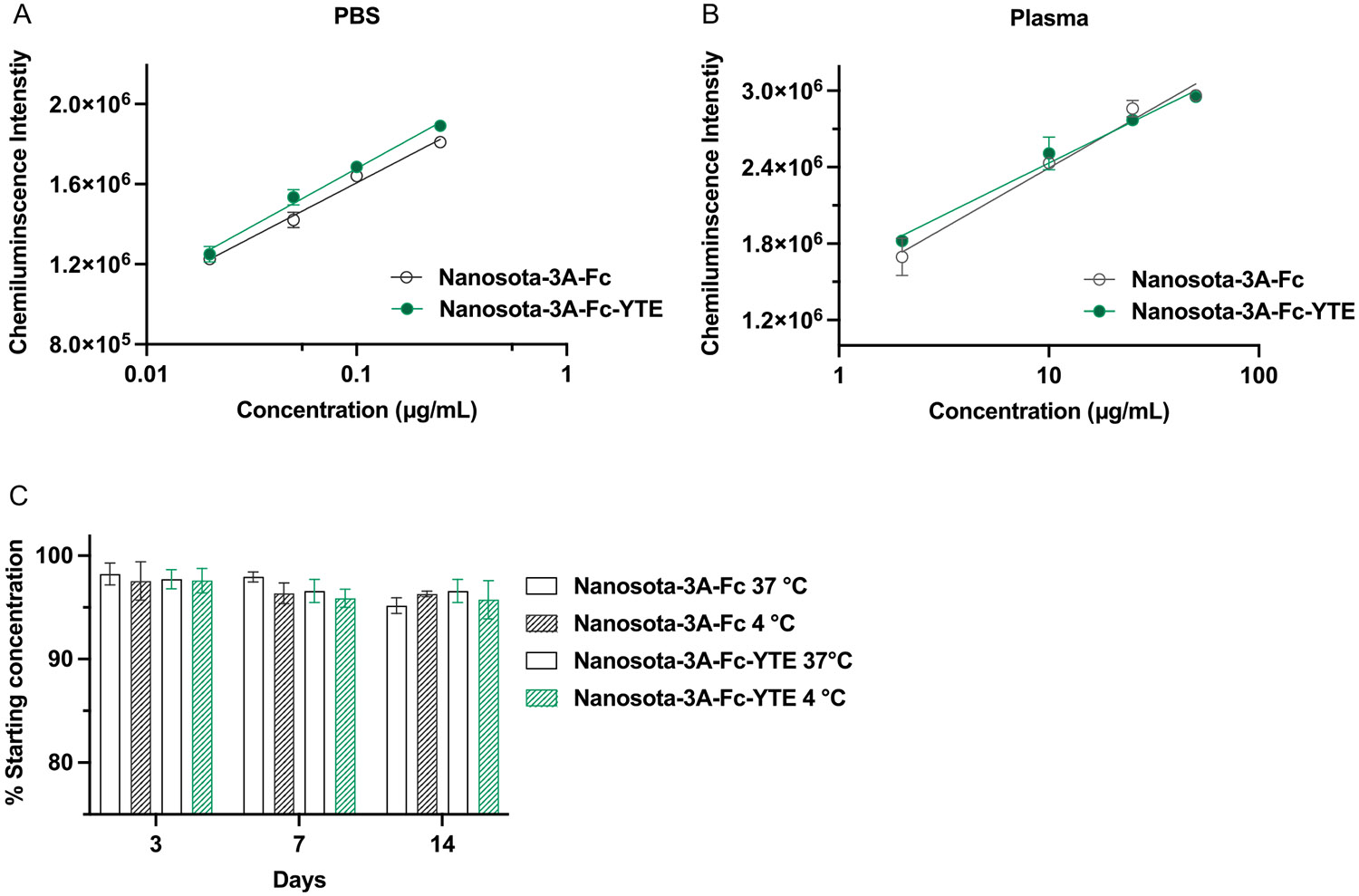
Quantification of Nanosota-3A-Fc and Nanosota-3A-Fc by ELISA and their storage stability. A) The log-linear standard curves for Nanosota-3A-Fc and Nanosota-3A-Fc-YTE in PBS have a quantification range of 0.02–0.5 μg mL^−1^ (*r*^2^ > 0.95). B) The log-linear standard curves for Nanosota-3A-Fc and Nanosota-3A-Fc-YTE in plasma have a quantification range of 2–50 μg mL^−1^ (*r*^2^ > 0.93). C) Nanosota-3A-Fc and Nanosota-3A-Fc-YTE can be stably stored in PBS (pH 7.4) at 4 or 37 °C for at least 2 weeks. Results are shown as the mean ± SD (*n* = 3) and are representative of three independent experiments.

To develop a robust and reproducible quantification methodology for Nanosota-3A fused with human IgG1 Fc or Fc-YTE, we first assessed the concentration-dependent binding of nanobodies (diluted in phosphate-buffered saline (PBS)) to SARS-CoV-2 spike RBD peptide that was immobilized onto 96-well plates. The RBD-captured Nanosota-3A fusion proteins were detected by horseradish peroxidase (HRP)-linked antihuman IgG1 Fc antibody, using a chemiluminescence-producing substrate to amplify the signals. There was a clear log-linear relationship between the Nanosota-3A fusion protein concentration and the chemiluminescence intensity (*r*^2^ > 0.95). The RBD binding curves of Nanosota-3A-Fc and Nanosota-3A-Fc-YTE were nearly identical (Figure 1A), indicating minimal impact of the Fc-YTE substitutions on the RBD binding of nanobody fusions. The concentration-dependent binding of Nanosota-3A-Fc and Nanosota-3A-Fc-YTE to SARS-CoV-2 spike RBD served as the mechanistic basis for this quantitative enzyme-linked immunosorbent assay (ELISA), and the quantification range for both fusion proteins was 0.02–0.5 μg mL^−1^ in PBS.

Furthermore, the standard curves for quantification of Nanosota-3A fusion proteins in plasma were also established (Figure 1B). To minimize the interferences caused by the high endogenous plasma protein levels, it was necessary to dilute the plasma samples by at least 100-fold in PBS prior to the ELISA. The quantification range for both fusion proteins was 2–50 μg mL^−1^ in plasma (r^2^ > 0.93).

### 2.2. Nanosota-3A-Fc and Nanosota-3A-Fc-YTE Exhibit Excellent Storage Stability

The stability of Nanosota-3A-Fc and Nanosota-3A-Fc-YTE in PBS was evaluated at 4 °C or 37 °C for 2 weeks. Under the sterile conditions at either temperature, there was less than 5% loss in the fusion protein concentration (Figure 1C), possibly owing to the protein adsorption, aggregation, and/or degradation, indicating excellent storage stability of both nanobody fusions in aqueous solution under ambient temperatures.

### 2.3. YTE Substitutions of Fc Domain Minimally Affect the Binding of Nanosota-3A-Fc to SARS-CoV-2 Spike Protein

**Figure 2.**
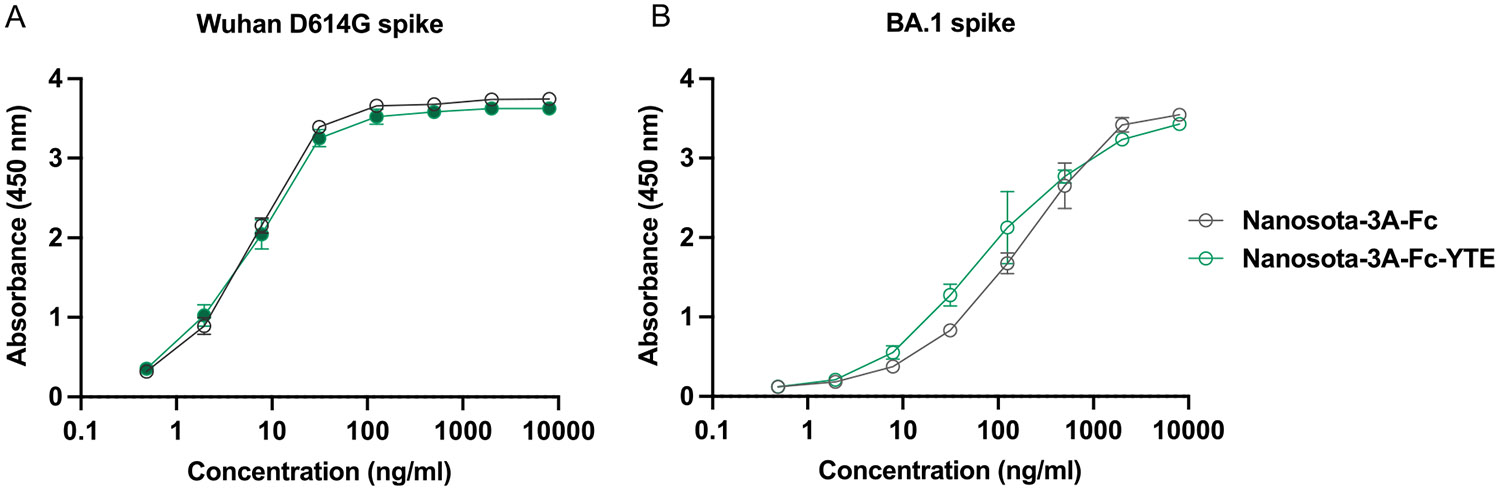
YTE substitutions of Fc domain minimally affect the binding of Nanosota-3A-Fc to SARS-CoV-2 spike protein. The binding of Nanosota-3A-Fc and Nanosota-3A-Fc-YTE to A) the Wuhan D614G spike and B) Omicron BA.1 spike was assessed by ELISA. The plates were coated with the respective spike ectodomain, incubated with Nanosota-3A-Fc or Nanosota-3A-Fc-YTE, and detected by the HRP-conjugated antihuman Fc antibody, which were measured for the absorbance at 450 nm. Results are shown as the mean ± SD (*n* = 3) and are representative of three independent experiments.

We have previously reported that Nanosota-3A-Fc has a high binding affinity for SARS-CoV-2 spike protein (K_d_ = 4.55 nM) and possesses potent neutralizing activity against both the prototypic Wuhan strain and Omicron BA.1 live viruses (IC_50_ = 29.5 pm).^[12]^ To verify whether Nanosota-3A-Fc-YTE retains the binding affinity for SARS-CoV-2 spike protein, we assessed the binding of a broad concentration range (from 0.5 ng mL^−1^ to 8 μg mL^−1^) of Nanosota-3A-Fc and Nanosota-3A-Fc-YTE to both the Wuhan D614G and the Omicron BA.1 spike ectodomains. As shown in Figure 2, the ELISA profiles (i.e., the absorbance at 450 nm) for Nanosota-3A-Fc and Nanosota-3A-Fc-YTE were statistically indistinguishable across the entire concentration range, indicating that the YTE substitutions of Fc domain minimally affect the binding of Nanosota-3A-Fc to either the prototypic Wuhan or Omicron BA.1 spike protein.

### 2.4. YTE Substitutions of Fc Domain Markedly Increase the Binding Affinity of Nanosota-3A-Fc for hFcRn at Acidic pH

**Figure 3.**
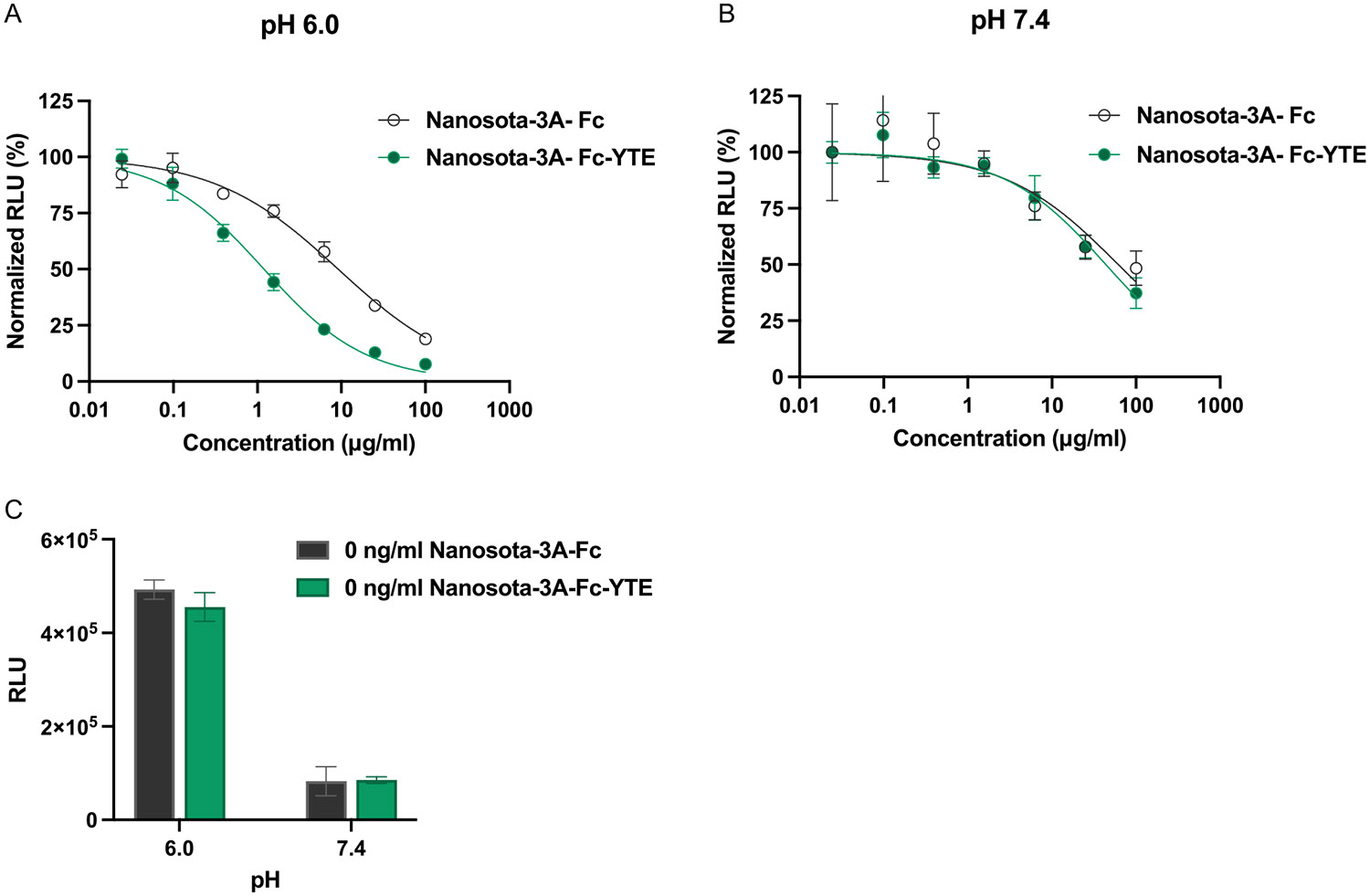
YTE substitutions of Fc domain markedly increase the binding affinity of Nanosota-3A-Fc for hFcRn at acidic pH. Concentration-dependent inhibition curves on IgG–hFcRn binding by Nanosota-3A-Fc and Nanosota-3A-Fc-YTE were obtained at A) pH 6.0 and B) pH 7.4. C) The maximum bioluminescent signals (RLU) were observed in absence of Nanosota-3A-Fc or Nanosota-3A-Fc-YTE, which were used to calculate the normalized RLU values in the presence of Nanosota-3A fusions. Results are shown as the mean ± SD (*n* = 3) and are representative of three independent experiments.

Mechanistically, the recycling efficiency of FcRn for IgG—the key determinant for the circulation persistence of IgG in vivo—is dictated by the binding affinity of FcRn for the Fc domain at acidic pH found in the endosomes of endothelial cells, relative to that at neutral pH maintained at the cell surface. To evaluate the effect of YTE substitutions on the hFcRn binding to nanobody Fc fusions, a NanoBiT-based competition immunoassay was employed, in which a specific and stochiometric binding interaction between hFcRn–SmBiT and human polyclonal IgG–LgBiT produces a bioluminescent signal (RLU), the presence of Fc fusion protein competes with IgG for hFcRn binding that reduces the bioluminescence intensity.^[17]^ AtpH 6.0, we obtained two IgG/hFcRn binding curves in the presence of ascending levels (0.02–100 μg mL^−1^) of Nanosota-3A-Fc and Nanosota-3A-Fc-YTE, respectively. There was clearly a leftward shift caused by Nanosota-3A-Fc-YTE (Figure 3A), indicating its increased hFcRn binding at pH 6.0 compared to the wild-type Fc counterpart. Based on the concentration-dependent reduction in the normalized RLU values, the IC_50_ was 1.2 μg mL^−1^ (15 nm) for Nanosota-3A-Fc-YTE and 9.2 μg mL^−1^ (118 nm) for Nanosota-3A-Fc, respectively, reflecting a nearly eightfold increase in the hFcRn binding affinity endowed by the YTE substitutions. By contrast, the IgG/hFcRn binding curves at pH 7.4 in the presence of Nanosota-3A-Fc and Nanosota-3A-Fc-YTE were indiscriminable, indicating the minimal impact of the YTE substitutions on hFcRn binding (Figure 3B). It is important to note that the maximum bioluminescent intensity (assigned as 100% normalized RLU, in the absence of nanobody Fc fusion protein) was fivefold lower at pH 7.4 than at pH 6.0 (Figure 3C), consistent with the fact that FcRn binds to the Fc domain with diminished affinity at neutral pH.^[18]^ Together, these findings provide strong evidence that the YTE substitutions greatly enhance the recycling efficiency of Nanosota-3A-Fc by hFcRn.

### 2.5. YTE Substitutions of Fc Domain Pronouncedly Prolong the Circulation Half-Life of Nanosota-3A-Fc

To determine whether the increased binding of Nanosota-3A-Fc-YTE to hFcRn at acidic pH can result in prolonged circulation half-life in vivo, we evaluated the plasma pharmacokinetics of Nanosota-3A fusion proteins in hFcRn transgenic mice (Tg32 strain), a well-validated preclinical model for evaluating the pharmacokinetics of human IgG mAbs.^[19]^

**Figure 4.**
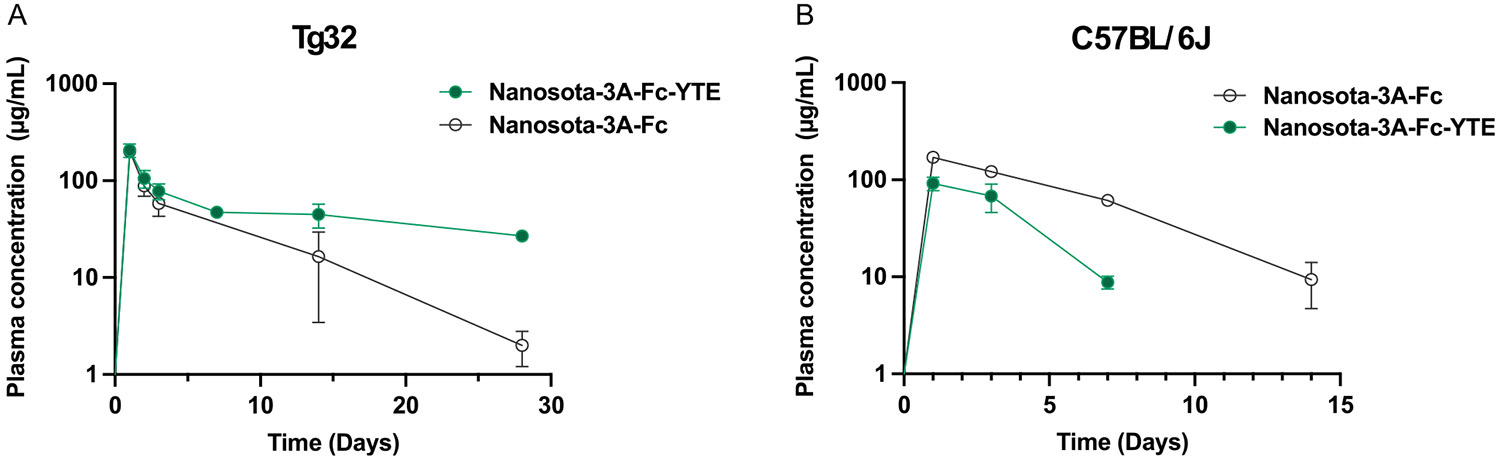
Plasma pharmacokinetics of Nanosota-3A-Fc and Nanosota-3A-Fc-YTE in mice. A) The hFcRn transgenic mice (Tg32, *n* = 6 per group) and B) the wild-type C57BL/6J mice (*n* = 4 per group) received a single intraperitoneal injection (20 mg kg^−1^) of Nanosota-3A-Fc or Nanosota-3A-Fc-YTE. A small volume of blood was collected from each mouse longitudinally at 24, 48, 72, 168, 336, and 672 h via the tail vein nicking, and the plasma drug concentrations were quantified by ELISA. Results are shown as the mean ± SD.

Following intraperitoneal injection, Nanosota-3A-Fc and Nanosota-3A-Fc-YTE were rapidly absorbed into the systemic circulation with nearly identical peak plasma concentration occurred at 24 h (Figure 4A). An initial rapid decline till 72 h reflects the distribution phase, during which the drug levels in the circulation drop quickly owing to the drug extravasate into the peripheral tissues. The subsequent elimination phase is driven by the drug removal from the body once the drug concentration in plasma and tissues has reached an equilibrium. The Nanosota-3A-Fc-YTE level persisted in plasma well above that of Nanosota-3A-Fc throughout the β-phase, indicating much reduced clearance of Nanosota-3A-Fc-YTE relative to Nanosota-3A-Fc. The elimination half-life of Nanosota-3A-Fc and Nanosota-3A-Fc-YTE was 5.1 days and 24.8 days, respectively, reflecting a 4.7-fold half-life extension caused by the YTE substitutions.

To further confirm hFcRn as a key determinant for the circulation half-life of Nanosota-3A Fc fusion, we also performed the pharmacokinetic studies in C57BL/6J mice—the wild-type background from which the Tg32 strain was derived—which express mouse FcRn (mFcRn). In striking contrast to the plasma pharmacokinetic profiles observed in hFcRn transgenic mice, Nanosota-3A-Fc-YTE was cleared from the circulation more rapidly than Nanosota-3A-Fc (Figure 4B). The half-life of Nanosota- 3A-Fc and Nanosota-3A-Fc-YTE in C57BL/6 mice was 2.9 days and 1.4 days, respectively. This is not unexpected, as mFcRn is known to bind to the human IgG1 Fc-YTE with a higher affinity relative to the wild-type Fc at both acidic and neutral pH,^[18]^ leading to reduced recycling of Fc-YTE-engineered IgG1 and consequently a shorter half-life in the wild-type mice. Taken together, these results demonstrate hFcRn-dependent half-life extension endowed by human IgG1 Fc-YTE fusion of Nanosota-3A.

### 2.6. Nanosota-3A-Fc-YTE Confers Durable Prophylaxis against SARS-CoV-2 Omicron Infection in Mice

**Figure 5.**
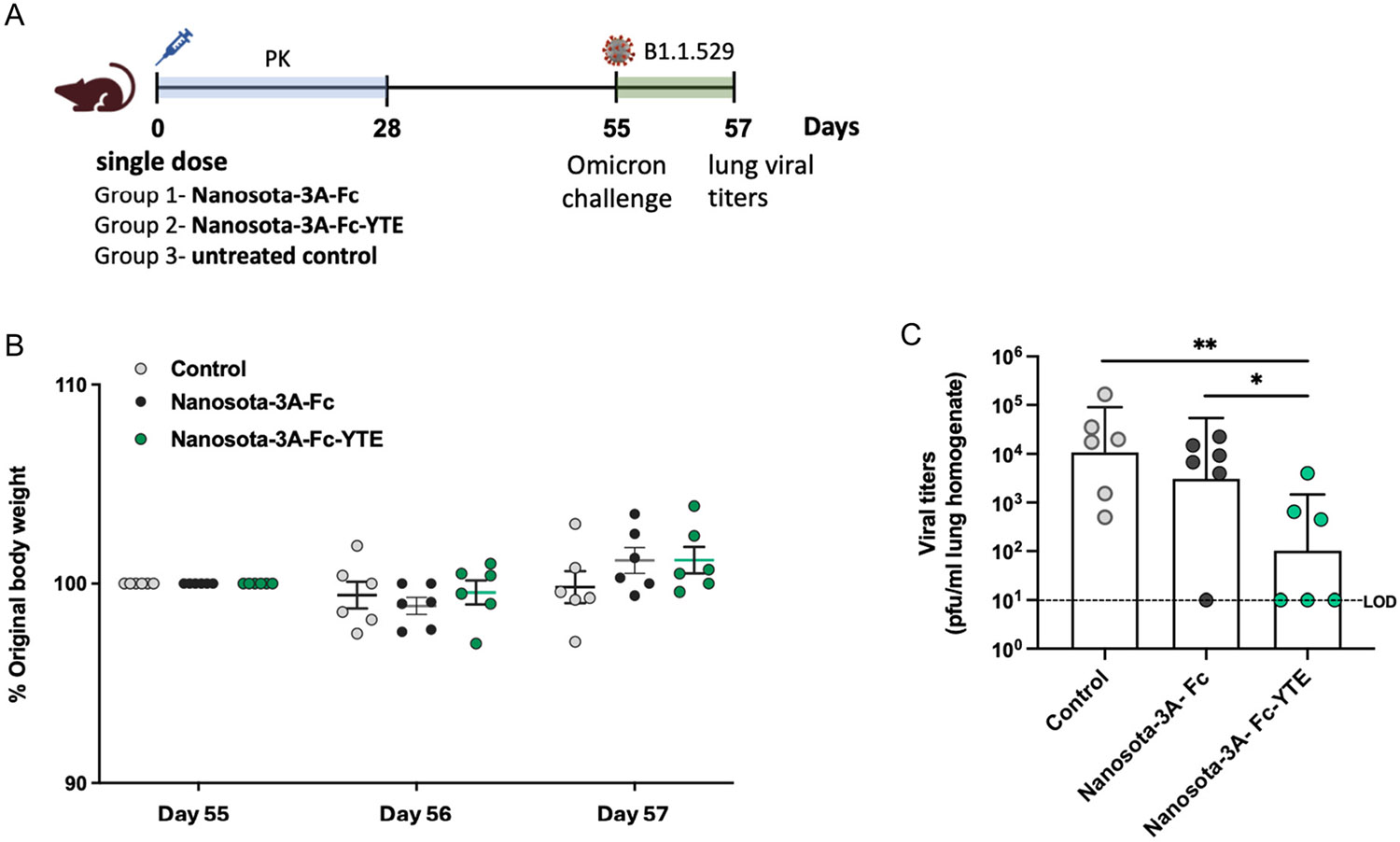
Nanosota-3A-Fc-YTE confers durable prophylaxis in mice exposed to SARS-CoV-2 Omicron. A) Overview of the experimental design. Following a single intraperitoneal dose (20 mg kg^−1^) of Nanosota-3A-Fc or Nanosota-3A-Fc-YTE on day 0, hFcRn transgenic mice (*n* = 6 per group) were evaluated for the plasma pharmacokinetics up to day 28, which were challenged with Omicron B.1.1.529 (10^5^ PFU) via intranasal inoculation on day 55. The mice were euthanized 2 days postinfection on day 57. B) The body weight of mice in each cohort was measured on day 55, 56, and 57. C) The viral titers in the lung homogenate supernatants were measured by a plaque assay. Results are shown as the geometric mean + SD (*n* = 6). The statistical differences between the respective groups were analyzed by one-tailed Mann–Whitney test; *p < 0.05; **p < 0.01.

Encouraged by superior circulation persistence of Nanosota-3A-Fc-YTE relative to Nanosota-3A-Fc in hFcRn transgenic mice, we explored the prophylactic efficacy against SARS-CoV-2 Omicron infection in these mice. The same two cohorts of hFcRn transgenic mice from the pharmacokinetic study, which received a single intraperitoneal dose (20 mg kg^−1^) of Nanosota-3A fusions on day 0, were challenged with intranasal exposure of Omicron B.1.1.529 on day 55. Two days postinfection (on day 57), the viral titers in the lungs were assessed (Figure 5A). During this short timeframe, no weight loss or clinical sign was observed (Figure 5B). Compared to the untreated control mice, the geometric mean of viral load in the lungs of mice receiving Nanosota-3A-Fc-YTE was reduced by 104.7-fold (*p* = 0.007) with 50% of the mice free of detectable virus infection (Figure 5C). Meanwhile, the Nanosota-3A-Fc-treated mice had 3.5-fold less viral titers than the untreated control mice, although the difference did not reach statistical significance (*p* = 0.41). Nanosota-3A-Fc-YTE exhibited significantly higher anti-Omicron protection than Nanosota-3A-Fc (*p* = 0.02). These results indicate that by the virtue of half-life extension, Nanosota-3A-Fc-YTE confers durable prophylactic efficacy superior to Nanosota-3A-Fc against Omicron infection.

## 3. Discussion

The advance in Fc engineering has led to the development of a new generation of IgG mAbs with improved circulation persistence.^[16,15c,20]^ Several SARS-Cov-2–neutralizing mAbs previously approved under the FDA EUA have incorporated amino acid substitutions in the Fc regions to extend their circulation half-lives.^[16,21]^ However, the application of such technology to nanobody therapeutics has not been reported previously. The present work aimed to bridge such a gap and explore Fc engineering to extend the circulation half-life of Nanosota-3A-Fc, a novel nanobody Fc fusion with potent neutralizing activity against SARS-CoV-2 Omicron BA.1 variant.

As one of the clinically validated strategies for the half-life extension of IgG mAbs, the YTE-substituted IgG1 Fc domain has been extensively investigated over the past two decades, culminating in the development of nirsevimab, the first FDA-approved, YTE-modified mAb that provides prophylaxis of respiratory syncytial virus in infants with a serum half-life of ≈70 days.^[22]^ Studies have demonstrated that relative to the wild-type human IgG1 Fc, the Fc-YTE domain binds to hFcRn with tenfold higher affinity under the acidic conditions without affecting its release at neutral pH.^[18]^ Through such pH-dependent FcRn binding enhancement, the increased recycling of YTE-modified mAb in the endothelial cells can result in twofold to fourfold halflife extension relative to that of the parental mAb in cynomolgus monkeys and humans.^[22b,23]^

When Nanosota-3A was fused with the human IgG1 Fc-YTE, its binding affinity for hFcRn at pH 6.0 was increased by eightfold compared to the wild-type Fc fusion, while the binding at pH 7.4 remained unaffected (Figure 3). In turn, this led to a prolonged plasma half-life of 24.8 days for Nanosota-3A-Fc-YTE in hFcRn transgenic mice, a 4.7-fold extension relative to that of Nanosota-3A-Fc (Figure 4). These findings are largely consistent with the literature on the pharmacokinetics of YTE-substituted mAbs,^[22a,23]^ strongly suggesting that Fc engineering with YTE substitutions can be broadly applicable to generate long-circulating nanobody therapeutics. The extended half-life of Nanosota-3A-Fc-YTE translated to long-lasting prophylactic efficacy against SARS-CoV-2 infection in mice. A single dose of Nanosota-3A-Fc-YTE (20 mg kg^−1^) administered nearly 2 months prior to Omicron B.1.1.529 virus challenge was able to reduce the viral titers by two orders of magnitude in the lungs, with half of the treated mice having no detectable viral infection. By contrast, Nanosota-3A-Fc under the identical experimental conditions exerted much less protection despite possessing equally potent antiviral activity in vitro. These results clearly demonstrate an inverse correlation between the nanobody half-life and the lung viral titers in Omicron-challenged mice, underscoring the circulation persistence as an essential determinant for long-term prophylactic efficacy of Nanosota-3A Fc fusion in vivo. Even though the plasma concentration of Nanosota-3A-Fc-YTE on day 55 was below the quantification limit (2 μg mL^−1^, 25.6 nm), the nanobody level in the lung is likely still above the antiviral IC_50_ value (2.3 ng mL^−1^, 29.5 pm). It is plausible that the prolonged circulation half-life of Nanosota-3A-Fc-YTE results in elevated and sustained nanobody exposure in the respiratory airways, neutralizing the virus entry into the alveolar epithelium and providing long-lasting protection against Omicron infection. Consistent with this notion, it has been previously shown in cynomolgus monkeys that the level of YTE-containing mAb in the bronchoalveolar lavage was elevated as a direct result of half-life extension.^[23]^ Furthermore, AZD7442 (a cocktail of tixagevimab and cilgavimab) contains two SARS-CoV-2–neutralizing mAbs with YTE substitutions that have extended half-lives of ≈90 days with 1–2% of serum AZD7442 detected in nasal mucosa.^[24]^

To our knowledge, this study is the first to extend nanobody half-lives by engineering the Fc region of IgG, enabling their use for pre-exposure prophylaxis against SARS-CoV-2 infection. Although similar approaches have been explored with conventional mAbs,^[16,21,24]^ prophylactic nanobodies offer distinct therapeutic advantages due to their unique properties.^[25]^ First, their single-domain antigen-binding structure allows them to access epitopes on the SARS-CoV-2 spike protein that conventional antibodies cannot reach, enabling unique antiviral mechanisms.^[26]^ Second, their small size allows for efficient intranasal delivery, offering a needle-free administration route.^[12]^ Third, their enhanced stability makes them more cost-effective to produce, transport, and store, improving accessibility.^[11e]^ Taken together, our findings open a new avenue for developing nanobody-based prophylactics against SARS-CoV-2 and other viral infections, combining strong efficacy with practical benefits.

## 4. Conclusion

The primary thrust of this work is to develop and evaluate Fc-engineered nanobody fusion for the pharmacokinetic enhancement. The durable protection from SARS-CoV-2 Omicron infection in mice conferred by a single dose of Nanosota-3A-Fc-YTE administered nearly 2 months prior to virus exposure demonstrates the promise of long-circulating nanobodies as powerful prophylactics. This study illustrates that Fc-YTE fusion of a nanobody could increase its circulation persistence without compromising the antigen specificity, thereby harnessing the full potential of this burgeoning therapeutic platform. For the first time, our work provides the proof of concept for exploring Fc-engineered nanobody fusions as promising next-generation prophylactics for emerging SARS-CoV-2 variants.

## 5. Experimental Section

### Production of SARS-CoV-2 Spike Ectodomain and RBD:

HEK293T cells stably expressing SARS-CoV-2 spike ectodomain (residues 14–1211) and RBD (residues 319–529) with a C-terminal His tag were generated as described previously.^[11e]^ The secreted proteins were harvested and purified on a nickle-nitrilotriacetic acid (Ni-NTA) column and then on a Superdex 200 Increase 10/300 gel filtration column (Cytiva).

### Production of Nanosota-3A-Fc and Nanosota-3A-Fc-YTE:

Nanosota-3A-Fc and Nanosota-3A-Fc-YTE genes were subcloned into lentiviral vetors that use cytomegalovirus promoter (Lenti-CMV). N-terminal tissue plasminogen activator signal peptide and C-terminal IgG1 Fc or Fc-YTE were fused to the nanobody sequence. Lentiviral particles were packaged using the nanobody-containing Lenti-CMV vectors and were used to infect HEK293F cells in the presence of puromycin (Gibco). The proteins were harvested from the supernatants of cell culture media, initially isolated by affinity chromatography with a Protein A column, and further purified by a Superdex 200 gel filtration column (Cytiva).^[12]^

### Quantification of Nanosota-3A-Fc and Nanosota-3A-Fc-YTE by ELISA:

ELISA was performed to detect the binding of nanobody fusion proteins to the SARS-CoV-2 RBD as previously reported.^[11e]^ The high-binding 96-well plates (Corning) were coated with recombinant SARS-CoV-2 RBD (1 μg mL^−1^,75μL) at 4 °C overnight. The plates were washed with PBS (pH 7.4) and blocked with 2 % BSA at room temperature for 1 h. The plates were then washed with PBST (PBS + 0.05% Tween, pH 7.4) and incubated with the nanobody fusion protein samples (either in PBS or in mouse plasma) at room temperature for 1 h. The plates were again washed with PBST and then incubated with HRP-conjugated goat antihuman-Fc antibody (Jackson ImmunoResearch,1:20 000 dilution) at room temperature for 1 h. Following sequential wash with PBST and PBS, the plates were incubated with SuperSignal Femto substrate (Thermo Fisher) for 1 min, and the chemiluminescence signal was measured using a BioTek Synergy H1 plate reader (Agilent).

### Stability of Nanosota-3A-Fc and Nanosota-3A-Fc-YTE:

Nanosota-3A-Fc or Nanosota-3A-Fc-YTE (200 μg mL^−1^ in sterile PBS, pH 7.4) was incubated at 4 or 37 °C for 2 weeks. On days 3, 7, and 14, samples were collected in triplicates and analyzed by ELISA. The concentration of Nanosota-3A-Fc or Nanosota-3A-Fc-YTE was determined according to the respective standard curves.

### Binding of Nanosota-3A-Fc and Nanosota-3A-Fc-YTE to the Prototypic SARS-CoV-2 and Omicron Spike Ectodomain:

An ELISA was performed to assess the binding of Nanosota-3A fused with the wild-type IgG1 Fc versus Fc-YTE, to the prototypic SARS-CoV-2 Wuhan D614G or the Omicron BA.1 spike.^[12]^ High-binding 96-well plates were coated with the prototypic or Omicron BA.1 spike ectodomain at 4 °C overnight. The plates were washed with PBST and then incubated with Nanosota-3A-Fc or Nanosota-3A-Fc-YTE. After washing with PBST, HRP-conjugated goat antihuman-Fc antibody was added to the plates (1:3000 dilution). After a final wash, the ELISA substrate TMB (Invitrogen) was added to yield a colorimetric response, which was stopped using 1N H_2_SO_4_. The plates were then read immediately for the absorbance at 450 nm using BioTek Synergy LX multi-mode plate reader (Agilent).

### Binding of Nanosota-3A-Fc and Nanosota-3A-Fc-YTE to Human Neonatal Fc Receptor (hFcRn):

The competitive binding of Nanosota-3A-Fc or Nanosota-3A-Fc-YTE to hFcRn in the presence of human polyclonal IgG1 was determined at pH 6.0 and pH 7.4 using Lumit FcRn Binding Immunoassay kit (Promega) according to the manual. The luminescence was measured using a Synergy plate reader. The normalized luminescence was calculated as the percentage of luminescence in the presence of nanobody Fc fusion relative to the maximum bioluminescent signal obtained in the absence of nanobody Fc fusion. Data were fitted to a four-parameter logistic regression equation to calculate IC_50_ (GraphPad).

### Pharmacokinetic Studies:

All animal procedures were conducted in accordance with the Guide for the Care and Use of Laboratory Animals of the National Institute of Health. The animal procedure protocols were approved by the Institutional Animal Care and Use Committee at the University of Tennessee Health Science Center (IACUC protocol number: 22-0371).

Human FcRn transgenic mice (B6.Cg-*Fcgrt^tm1Dcr^* Tg(FCGRT)32Dcr/DcrJ; Tg32) were purchased from The Jackson Laboratories. The mice were randomized into two groups (six per group, equal number of females and males, 6 weeks old) and were injected intraperitoneally with Nanosota-3A-Fc or Nanosota-3A-Fc-YTE (20 mg kg^−1^). At 24, 48, 72, 168, 336, and 672 h, a small volume of blood was collected from each mouse via the tail vein nicking, and the plasma samples were stored at −80 °C till analysis.

In a separate experiment, C57BL/6J mice (Jackson Laboratories) were randomized into two groups (four per group, equal number of females and males, 6 weeks old) were intraperitoneally injected with Nanosota-3A-Fc and Nanosota-3A-Fc-YTE (20 mg kg^−1^). At 24, 48, 72 and 168 h, a small volume of blood was collected from each mouse via the tail vein nicking, and the plasma samples were stored at −80 °C till analysis.

The plasma concentration of Nanosota-3A-Fc and Nanosota-3A-Fc-YTE was determined by ELISA. The quantification of plasma concentrations was based on the standard curves as described above, which were included in each ELISA experiment. All plasma samples were analyzed in triplicates.

### SARS-CoV-2 Omicron B.1.1.529 Challenge:

Vero E6 (ATCC, CRL-1586) or Vero-TMPRSS2 cells^[27]^ were maintained in minimum essential medium (MEM) with Dulbecco’s or Earle’s salts with L-glutamine, 1% penicillin/ streptomycin, and 10% heat-inactivated fetal bovine serum (FBS), i.e., the complete MEM. Cells were not grown beyond passage 30. SARS-CoV-2 c (SARS-CoV-2/human/USA/COM-121 721-02/2021, SJ# C2364) was a kind gift of Dr. Richard Webby, St. Jude Children’s Research Hospital. SARS CoV-2 Omicron B.1.1.529 was grown in Vero-TMPRESS2 cells cultured in Dulbecco’s modified Eagle medium (DMEM) with 2% FBS and 1 mg mL^−1^ geneticin, and the viral titer was measured by a plaque assay as we reported previously described previously.^[28]^ Virus seed stocks were frozen in 0.5 mL aliquots and kept at −80 °C until used. All cell culture reagents were purchased from Fisher Scientific. All experiments were conducted within the BSL-3 or ABSL-3 facilities of the University of Tennessee Health Science Center Regional Biocontainment Laboratory (RBL) compliant with protocols that were reviewed and approved by the Institutional Biosafety Committee and the Institutional Animal Care and Use Committee.

On day 55, both groups of hFcRn transgenic mice from the previous pharmacokinetic study were exposed to SARS CoV-2 Omicron B.1.1.529 (10^5^ PFU, 25 μL per nare) via intranasal inoculation in the RBL ABSL3. Another group of age- and gender-matched naïve hFcRn transgenic mice (*n* = 6) as the untreated controls were infected under the identical conditions. Clinical signs and weights were recorded daily. All mice were euthanized 2 days postinfection on day 57. The lung homogenate supernatants were prepared, serially diluted in DMEM, and used to measure the viral titers in the lungs by a plaque assay using Vero E6 cells as described previously.^[28]^

### Statistical Analysis:

The viral titers in the lungs of the untreated and treated mice were used to calculate the geometric mean + SD (*n* = 6) of each group. The statistical differences between the respective groups were analyzed by one-tailed Mann–Whitney test (GraphPad); the p-value of less than 0.05 was considered statistically significant.

## Data Availability

The data that support the findings of this study are available from the corresponding author upon reasonable request.
